# Development of a Web-Based Intervention Course to Promote Students’ Well-Being and Studying in Universities: Protocol for an Experimental Study Design

**DOI:** 10.2196/23613

**Published:** 2021-03-09

**Authors:** Henna Asikainen, Nina Katajavuori

**Affiliations:** 1 Faculty of Educational Sciences University of Helsinki Helsinki Finland

**Keywords:** approaches to learning, psychological flexibility, well-being, online intervention tool, peer support, reflection

## Abstract

**Background:**

The decline in the well-being among university students well as increasing dropouts has become a serious issue in universities around the world. Thus, effective ways to support students’ well-being and their ability to study are highly needed.

**Objective:**

The purpose of this study was to build an intervention course for university students, which promotes both students’ well-being as well as their learning and study skills, and to describe the experimental study design that explores the effects of this intervention course.

**Methods:**

Research has shown that psychological flexibility has a great effect on the well-being as well as the study skills of students pursuing higher education. The basis of our intervention course was to promote psychological flexibility and students’ study skills with the help of peer support and reflection.

**Results:**

This course was offered as a voluntary course to all the students at the University of Helsinki twice during the academic year 2020-2021. The first course was from October to December and the second course was from January to March. This course was advertised in fall 2020 through social media and by different student organizations and program leaders at different faculties of the University of Helsinki. As of October 2020, we enrolled 566 students comprising 310 students for the course in fall 2020 and 256 students for the course in spring 2021. Of the 256 students who enrolled in the second course, 170 students voluntarily participated in this study and they answered the questionnaires, including all the measures, simultaneously with the participants in the first group and thus served as the control group. The effect of this course will be measured with multiple data, including questionnaire data, reflective journals, and physiological data of well-being with a longitudinal experimental design. This research very strictly follows the ethical guidelines drawn up by the Finnish National Board on Research Integrity. We expect to publish the results of this study in fall 2021 at the latest.

**Conclusions:**

We argue that a web-based, 8-week intervention course, which promotes both student well-being and their study skills, is a good way to support students pursuing higher education, and both aspects should be considered when supporting university students.

**International Registered Report Identifier (IRRID):**

DERR1-10.2196/23613

## Introduction

### Brief Overview of This Study

The decline in the well-being of university students and increased mental disorders experienced by students pursuing higher education have become a serious issue around the world [1-3]. In the United States, over 50% of the college students have a psychiatric disorder and over 60% have experienced serious anxiety [4]. In Finland, one-third of the students have experienced mental problems [5]. At the same time, there has been an increase in student dropout rates and study times have increased. As in most European countries, in Finland, 3 important aims to solve this problem have been added to the agenda: students’ completion of degrees, completion in a reasonable period, and reducing student dropout [6]. In addition, the demands of today’s workforce life require excellent life-long learning skills in students and the ability to solve complex and multidisciplinary problems under heavy workloads and stress. Thus, there is a huge discrepancy between the demands set for students and students’ well-being. Obviously, there is a need for services that would enhance students’ well-being, especially in the initial stages of studying due to the transition challenges being faced [7]. A growing body of evidence demonstrates that social and emotional skills greatly affect academic performance [8] and students’ studying and learning are related to their well-being [9]. Furthermore, there is evidence that many students encounter troubles in their learning and studying processes [10,11], and in higher education, the emphasis should also be paid to supporting students by considering both their well-being as well as their study skills and life-long learning skills [12]. Thus, effective ways to support students’ well-being and their ability to study are highly needed. Our aim was to describe a course that was developed to support students’ well-being as well as their study skills at university and to describe the plan for how to explore the effects of this course on students’ learning and well-being. This course was based on fostering students’ psychological flexibility and study skills, and peer support and reflection were chosen as the central pedagogical tools to support the development of these aspects.

### Theoretical Background

Well-being is not easy to define because there are many definitions and traditions and things to consider when thinking about well-being [13]. One prominent model of well-being defines well-being as having the following 3 parts [14]: emotional, psychological, and social well-being. Emotional well-being can be described as positive emotions toward life or good satisfaction in life. Psychological well-being relates to how individuals view themselves as functioning in life and it can be conceptualized though processes such as self-acceptance, sense of mastery and competence, positive relationships with others, feeling of personal growth or development, sense of goal-directedness in life, and autonomy [15]. Social well-being also refers to positive functioning but from a social perspective and it can be understood from 5 dimensions: social coherence, social acceptance, social actualization, social contribution, and social integration [16]. In this course, we aim to promote all 3 aspects of well-being, namely, emotional, psychological, and social well-being by practicing and developing one’s psychological flexibility.

Psychological flexibility describes people’s ability to be connected with the present and to regulate their emotions and actions despite the unpleasant feelings or thoughts they might have [17-19] and further, to take value-based actions. People with high psychological flexibility act according to their own values and accept their negative thoughts, emotions, and sensations rather than avoid them and deal with these negative emotions and thoughts by opening up to them and observing them from another perspective mindfully [20]. The origin of psychological flexibility lies in acceptance and commitment therapy (ACT) [17,18] and is based on the ACT theory of psychopathology. Promoting psychological flexibility has been shown to improve all aspects of well-being: emotional, psychological, and social well-being [18,21]. It is related also to physical well-being as it is negatively related to experiences of, for example, sleeping problems [22] and eating disorders [23]. Research and meta-analyses have shown that psychological flexibility can reduce depression and anxiety [24,25] and has been found to play a central role in stress management [26] and life management [27], low quality of life, stress management and well-being [28,29], as well as self-compassion [30]. It has also been shown to have a central role in improving performance, well-being, and results in the workplace [31,32]. Some ACT-based interventions have been made in higher education and they have shown to be successful in improving students’ well-being and stress levels [21,32-35] as well as their psychological flexibility [21]. Furthermore, ACT-based web-based interventions have been found beneficial in a meta-analysis of comparison between face-to-face and web-based interventions as there were no differences in the effectiveness, and thus, strong support has been brought up for the adoption of web-based psychological interventions [36].

Psychological flexibility is established through 6 overlapping core processes, which are strengthened in ACT. These core processes are acceptance, cognitive defusion, being present, self as context, values, and committed action [18]. Acceptance is the opposite of experimental avoidance and means “the active embrace of those private events occasioned by one’s history without unnecessary attempts to change their frequency of form” [18]. Acceptance is not a matter of tolerance but rather, it supports value-based actions and exploration of feelings, memories, and thoughts from an observer perspective [37]. Cognitive defusion represents the process or techniques through which one’s relationship to these negative thoughts is altered and the ability to look at one’s own thoughts as separate parts of internal behavior and not consider them to be truth about the world or oneself [38]. Being present relates to continuous contact with a range of events or thoughts as they occur, emphasizing the ongoing process of defused and nonjudgmental description of thoughts [18]. Being present comprises seeing oneself as a context or a container of one’s experiences and thoughts, and thus, seeing these thoughts as being separate from the self [17]. Values are the foundation for fostering psychological flexibility, and value-based action and behavior are necessary to life satisfaction and experience of a meaningful life [36,39]. Values are also an important part of psychological flexibility as they offer guidance in terms of what behaviors are likely to lead to long-term satisfaction and experience of meaningfulness in life, which is an explicit aim in ACT [18]. The sixth aspect of flexibility, that is, committed action, leads to a value-emphasized life through taking value-based actions, instead of actions motivated by avoidance of negative thoughts [18]. Psychological flexibility has also a big role in university studies. It has been shown to be positively related to positive emotions in learning [40], integration into studying [41], self-regulation [42,43], and study progression [39,40]. Recent pilot studies have indicated that the theory of psychological flexibility is the core process in explaining procrastination in the higher education context [43,44] and it has also been shown to be particularly important for students who are at higher risk of academic failure [45]. Thus, the importance of psychological flexibility is evident in the university context. For this reason, all its 6 processes will be promoted and practiced in our course in order to foster the development of psychological flexibility.

In addition to psychological flexibility, research has shown that the deep approach to learning, that is, deep level processing in meaningful learning, is related to better learning outcomes [46,47]. However, recent research has pointed out that the deep approach to learning is not enough if the students are not organized in their studying [11,48]. Thus, the role of organized studying in significant in successful studying at a university [49-51]. Organized studying includes good time-management and self-regulation skills. Research has shown that it is possible to promote students’ time-management skills through interventions in which students learn and practice organizational skills [52,53]. Furthermore, research has shown that students’ approaches to learning are related to students’ well-being at the university, thereby showing that poor study skills can lead to a risk of study-related burnout in studies [9] and further, time allocation to important activities is a central part in achieving a meaningful life [54]. For these reasons, it would be important to support students’ time-management skills in order to improve their studying as well as to help students to allocate time better to things that are important to them. As deep approach to learning is related to better achievement as well as better well-being, it is utmost important to enhance both students’ time-management skills as well as their deep-level learning.

In summary, both psychological flexibility and study skills, including good time-management skills, are needed to foster university students’ well-being and studying. Thus, our intervention course is based on practicing both these aspects. Two central tools to foster these aspects during the course are reflection and peer support. Reflection has a central role during this course because reflection supports learning, and deeper and critical reflection is related to deeper learning, and further, to better learning outcomes [55]. In addition, research has shown that peer support is related to successful study progression and it is a central factor to enhancing studying at the university [51]. Peer support has also been found to be important in increasing well-being [56].

Our aim was to develop a course to support students in being successful in their studies by supporting different aspects of well-being (physical, emotional, social, and psychological) as well as their study skills and further, to study the effects of this course. The central process to support these aims is to support the development of psychological flexibility as it has been shown to have positive effects on all aspects of well-being as well as learning and studying in higher education. We will now describe the course in more detail and the experimental study design with a control group for studying the course.

## Methods

### Intervention Course

An optional 8-week ACT-based web-based course was developed on the Moodle web-based platform and was completed online. This course was designed so that students do weekly assignments independently, reflect the themes of the course in small groups, and give and receive peer feedback of each other’s assignments. The course is suitable for Bachelor and Master level students and can be easily implemented to curricula. The teacher’s role in this course is to facilitate students’ progress during the course and monitor the group discussions and students’ assignments. The teachers also meet the students online in the course and give video instructions to the module’s themes and assignments. The teachers need to be familiar with the process of psychological flexibility as well as study skills to guide students properly and thus, training has been given to the teachers.

### Modules of the Intervention Course

**Figure 1:**
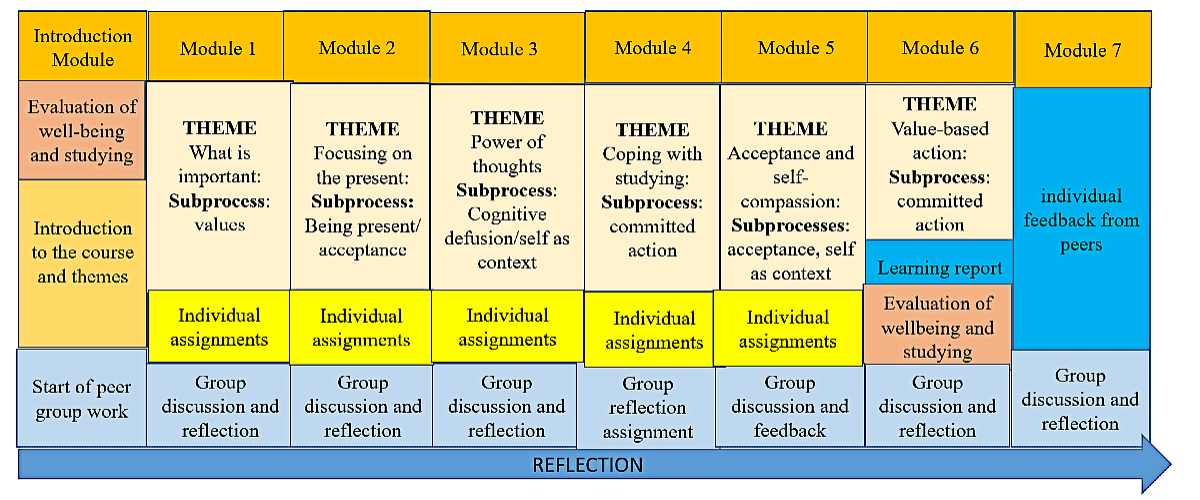
The design of the course.

This intervention course consists of 8 modules, each module lasting for 1 week (Figure 1). The course was developed to last for 1 study period. In the University of Helsinki, there are 4 study periods in 1 academic year, each period lasting for 8 weeks. Modules support the development of psychological flexibility and study skills. Each module focuses on to 1 or 2 processes of psychological flexibility. The processes are parallel to each other [17] but some processes are emphasized more in some of the modules. Each module includes video and text introductions to the themes of the week—individual experimental and reflective assignments—in which participants are asked to write down their experiences and reflections. In addition, small group discussions about the themes are held every week. We have developed introduction videos and materials for every week and part of the exercises and the assignments ourselves, but the exercises targeted to improve psychological flexibility are based on the work that has been done by Hayes [18] and a Finnish ACT psychologist Arto Pietikäinen who has also helped developing the course. Next, we present these modules in more detail.

#### Introduction Module

The Introduction module includes video introductions to the course and an introduction to the central theme of the course: psychological flexibility and well-being. This module includes all the practical information about course assignments and completing the course, including deadlines and guidelines for group work and giving peer feedback. To help students reflect on their well-being and studying, students evaluate their level of well-being and study skills with validated research instruments at the beginning of the course and receive web-based feedback on these evaluations. For example, students receive feedback about their risk of study-related burnout and receive feedback and guidance according to the results they receive. The same evaluations are also completed at the end of the course. The aim of the evaluations is to stop students to consider their well-being in more detail right at the beginning of the course, and further, to help them reflect the change in their evaluations during the course. Students are also offered to do simple exercises on promoting psychological flexibility and they start a time-management assignment in which they are asked to monitor and record their time usage for a week. The aim of this task is to help students become aware of their time usage and help them in the forthcoming assignments during this course. The time-management task is used to help students to develop their studying by helping them to improve their time-management skills, setting goals, and to allocate time to important things in life. In addition, the students also start the group work by introducing themselves to the peer group and sharing thoughts about the start of the course.

#### First Module

The theme of the *first* *module* is *What is important.* This module consists of materials and exercises related to one’s values. First, introductions to values and why they are important to consider are represented. In addition, students do exercises that help them to think about and clarify issues that are important to them in their life. For example, students are asked to think about themselves far into the future having lived a very good life and are asked to think about the things they could remember about their lives and what they valued. In addition, students are asked to think about several areas of life such as well-being, family, friends, and to think about what is important for them and what they could do to foster this value. After these exercises, students set a value-based goal that they would want to achieve, and this goal is revisited and monitored throughout the course. To help the students reflect on how their values are manifested in their lives, students have a group discussion where they reflect their thoughts about the time-management task and how time management could help with the achievement of the goal.

#### Second Module

The theme of the *second module* is *Focusing on the present*. This module focuses on practicing 2 subprocesses: being present and acceptance. In this module, introductions are given to what being present really means, how it affects our brain and learning, and why it is important. The exercises include mindfulness exercises, which also include practice of acceptance of bodily emotions and thoughts. In the exercises, students are, for example, asked to do something mindfully by paying attention to this situation (waiting for a bus, eating, etc) or students are instructed to be intentionally present and listening when communicating with a peer. This module also includes breathing and relaxation exercises. Students are asked to test these different kinds of exercises during the day and monitor how these exercises affect their well-being. A small group discussion is also included where students are asked to share their experiences of the exercises and how these exercises have affected their studying and well-being and could help them achieve their goals.

#### Third Module

The *third module* is related to the theme *Power of the thoughts.* This is related to 2 of the central processes of psychological flexibility, namely, cognitive defusion and self as context. First, introductions are given to thoughts, how powerful they are, how they are related to emotions, and why it is important to become conscious of one’s thoughts. With different exercises, students are encouraged to become conscious of their thoughts, to explore and test these thoughts, and to look at their thoughts just as thoughts and not facts or truths about themselves or the current situations. For example, students are instructed to think about their negative thoughts just as thoughts that are like floating leaves on a flowing river: they can be observed and they come and go. In addition, students are asked to find new alternative thoughts and think of the long-term effects of these different thoughts to their behavior and well-being. These exercises aim to help the students to change their relationship with the negative thoughts they might have. Students share their experiences and discoveries in small groups and further, they are asked to think of how becoming aware and accepting their thoughts can help them achieve their goals regarding studying and well-being. During this week, students also meet the teachers of the course online. The aim of this meeting is to discuss about the themes and questions of the course together with students and practice together to recognize some negative thoughts students might have related to situations in studying.

#### Fourth Module

The *fourth module* consists of exercises that are comprehensively related to the theme *Coping with studying.* This module focuses more on the studying ability, including study processes, general well-being, and on values and committed actions. This module consists of introductions to this theme, web-based lectures and assignments regarding studying ability, time management, and studying techniques. The significance of physical exercise, sleep, and nutrition in studying is also discussed to make studying and learning more effective. During this module, students are encouraged to think of their life habits (including sleeping, exercise, and nutrition) in order to reinforce the value-based actions related to these themes by testing and monitoring the effects of different exercises. Students are also asked to identify the issues and elements that contradict the student’s own values related to studying or general life habits. Students practice study techniques, which support deeper understanding in their studying. Students are encouraged to apply these techniques to their learning and studying and to monitor which techniques and which practices would work best for them. During this week, students discuss in their peer groups about their experiences about that week’s topics.

#### Fifth Module

The *fifth module* is related to the theme *Acceptance and self-compassion,* concentrating on subprocesses of acceptance and self as context. In this module, students continue with exercises and techniques, which help them to accept, confront, explore their thoughts, and concentrate on self-compassion exercises. Students are, for example, asked to monitor how they speak to themselves, what kind of thoughts they have toward themselves, and to practice compassion toward themselves. Students are asked to have a thankful attitude toward their lives and practice compassion toward their peers, for example, they are encouraged to do small actions such as calling a relative, which support their own values. In addition, they are asked to observe how this kind of behavior affects their well-being and studying. These exercises present common humanity of self-compassion. Finally, students are asked to reflect on these exercises in their peer groups and to think how these exercises affect their well-being and studying.

#### Sixth Module

The *sixth module* is related to the subprocess of *Committed actions.* This module consists of introduction to this theme and web-based lectures about the significance of committed value-based actions. Students are encouraged to become conscious of obstacles that hinder them to doing what is important to them and further, they practice engaging in actions that help them to achieve their goals, which are set up in the beginning of the course. Students write a final learning journal, in which they reflect on their learnings and experiences of the effects of the course on their studying and well-being, and further, they analyze how they have proceeded with the goal that they have set up in the beginning of the course. Furthermore, students evaluate their well-being and studying based on the questionnaires again and are encouraged to analyze the changes in their well-being and studying during the course. At the end of the week, students discuss in their peer groups about their experiences and ideas of how to enhance taking value-based committed actions.

#### Seventh Module

The *seventh module* is a *Concluding module*. Students provide individual constructive feedback of the reflective journals to 2 other students anonymously, and students have a final peer group discussion where they share their experiences of the course, including which exercises have been the most effective to them and why.

## Results

The effect of this course will be measured with multiple data, and informed consent will be collected in the beginning of the course. First, questionnaire data measuring psychological flexibility [57], stress [58], social, psychological, and emotional well-being [59], study-related burnout [60], and study skills [61] will be collected before, after, and 1 year after the course. These questionnaires are also the basis of students’ own evaluations in the beginning and at the end of the course. Students also receive feedback from these evaluations. Hierarchical linear modeling with full information maximum likelihood estimation will be used to examine changes over time. In order to test the impact of the self-assessment from premeasurement to postmeasurement, the Group×Time interaction will be explored. Second, qualitative data of the effects of the course will be analyzed comprising students’ reflective journals of the course and open-ended responses about their experiences of the course in the questionnaire, which are used at the end of the course. These experiences will be analyzed for the effects of the course by using inductive content analysis [62]. In addition to self-reported data, physiological data of well-being will be used to measure changes in their well-being. The Moodmetrics smart ring, which measures electrodermal activity or level of arousal [63], will be used to measure participants’ stress levels and how this stress level changes during the course compared to that in the control group. Recent research has found that Moodmetrics has been found to be a robust measurement of the stressfulness in the workplace [64]. Students will keep the ring on during the whole course, and changes in stress levels will be analyzed and compared between the control and experimental groups by using methods of longitudinal analysis. Furthermore, we will also combine self-reported and biophysical data to compare the effects of the intervention from different data.

This course was offered as a voluntary course to all the students at the University of Helsinki twice during the academic year 2020-2021. The first course was from October to December and the second course was from January to March. This course was advertised in fall 2020 through social media and by different student organizations and program leaders at different faculties of the University of Helsinki. As of October 2020, we enrolled 566 students comprising 310 students for the course in fall 2020 and 256 students for the course in spring 2021. Of the 256 students who enrolled in the second course, 170 students voluntarily participated in this study and they answered the questionnaires, including all the measures, simultaneously with the participants in the first group and thus served as the control group. This research very strictly follows the ethical guidelines drawn up by the Finnish National Board on Research Integrity [65]. We expect to publish the results of this study in fall 2021 at the latest.

## Discussion

Our aim was to develop a pedagogically reasonable and beneficial web-based course to support students’ well-being as well as their study skills at university. We designed this intervention course to equip students with tools to enhance their well-being and to help them develop their study skills because well-being should also be taken into account when fostering students’ learning and studying [9,45]. This course was based on the idea of fostering psychological flexibility, which has convincingly been shown to be an essential factor in improving psychological, social, and emotional as well as physical well-being in many ways [25,35,66,67]. We will analyze the effects of this course with multiple data comprising quantitative, qualitative, and biophysical data in order to obtain comprehensive data of the effects.

During this course, each subprocess of psychological flexibility is systematically trained during the modules. Study processes and study skills are trained especially during 2 modules; however, studying is reflected throughout the course. We have implemented effective pedagogical tools to foster students’ learning during this course, namely, reflection and peer group working. Thus, we expect to see positive effects on students’ well-being and study processes during this course. A very small pilot study comprising similar elements showed that this kind of course can have many positive effects on students’ well-being and study skills [68]. That pilot study was analyzed with only 20 students and had no control group, but open-ended experiences of the students indicated that the students had a positive attitude toward the course and that the course had a positive effect on their well-being and study skills [68]. We expect to obtain the preliminary data of this course in spring 2021.

## Abbreviations

ACT: acceptance and commitment therapy
